# Epidemiologische Ansätze zur Klärung wichtiger Forschungsfragen zu COVID-19 – eine Übersicht

**DOI:** 10.1007/s00103-021-03378-x

**Published:** 2021-07-13

**Authors:** Hajo Zeeb, Wolfgang Ahrens, Ulrike Haug, Linus Grabenhenrich, Iris Pigeot

**Affiliations:** 1 Leibniz-Institut für Präventionsforschung und Epidemiologie – BIPS, Achterstr. 30, 28359 Bremen, Deutschland; 2 Wissenschaftsschwerpunkt Gesundheitswissenschaften, Universität Bremen, Bremen, Deutschland; 3 Fachbereich Mathematik und Informatik, Universität Bremen, Bremen, Deutschland; 4 Abteilung Methodenentwicklung und Forschungsinfrastruktur (MF), Robert Koch-Institut, Berlin, Deutschland

**Keywords:** Epidemiologie, Forschungsbedarf, Pandemie, Studiendesigns, Epidemiology, Research needs, Pandemic, Study designs

## Abstract

Die Epidemiologie als wissenschaftliche Disziplin ist prädestiniert dafür, Kernfragen der COVID-19-Pandemie zu bearbeiten. Hierzu werden klassische und neue Methoden eingesetzt, es stellen sich jedoch auch neue Herausforderungen.

Der Beitrag bezieht sich auf die verschiedenen Phasen des bevölkerungsbezogenen Verlaufs der SARS-CoV-2-Infektion und COVID-19-Erkrankung. Basierend auf einer selektiven Literaturrecherche werden Beispielfragestellungen anhand von in Deutschland und international durchgeführten Studien vorgestellt und die jeweiligen epidemiologischen Ansätze diskutiert, aber auch Forschungslücken beschrieben.

Wissenschaftliche Fragen, die mit epidemiologischen Daten und Forschungsansätzen zu beantworten sind, stellen sich in jeder Phase des Infektions- und Krankheitsgeschehens. Beschreibende Daten werden vielfach über (wiederholte) Querschnittsstudien generiert. Für analytische Fragestellungen etwa zur Identifikation von Risikogruppen hätten besonders in der frühen Phase der Pandemie Fallkontrollstudien wertvolle Ergebnisse liefern können, wurden aber selten durchgeführt. Daten der Krankenkassen kommt eine wichtige Funktion in der Analyse von Verläufen zu; das Potenzial dieser Datenquelle in Bezug auf Fragestellungen zur Impfung kann jedoch vermutlich kaum genutzt werden. Eine verbesserte Koordination der diversen Studien sowie eine stärker auf frei zugängliche Daten (*Open Data*) ausgerichtete Forschungsinfrastruktur können den Beitrag der Epidemiologie zur Kontrolle dieser und zukünftiger Pandemien weiter stärken.

## Abstract (en)

Epidemiology as a scientific discipline is predestined to address key problems in the COVID-19 pandemic. In order to do so, classic and new methods are used, and new challenges are emerging.

This paper addresses the various phases of the population-based progression of SARS-CoV‑2 infection and COVID-19. Based on a selective literature search, sample questions from studies conducted in Germany and internationally are presented, their respective epidemiological approaches discussed, and research gaps described.

Scientific questions to be answered with epidemiological data and research approaches arise in every phase of infection and disease. Descriptive data are often generated via (repeated) cross-sectional studies. For analytical questions, such as the identification of risk groups, case-control studies could have provided valuable results, especially in the early phase of the pandemic, but were rarely conducted. Data from health insurance companies have an important function in the analysis of the course of disease; however, the potential of this data source with regard to questions on vaccination can probably hardly be used. Improved coordination of the various studies and a more “open data” oriented research infrastructure can further strengthen the contribution of epidemiology to the control of the current and future pandemics.

## Einleitung

Die Epidemiologie bietet die wesentlichen wissenschaftlichen Methoden für die Analyse der Entwicklung und des Verlaufs von Epidemien und Pandemien, zur Identifikation von Ansatzpunkten für Prävention sowie zur Untersuchung der Wirksamkeit von Präventions‑, Kontroll- und klinischen Maßnahmen. In der COVID-19-Pandemie muss die epidemiologische Forschung qualitative und aussagekräftige Informationen unter erheblichem Zeitdruck als Entscheidungsgrundlage bereitstellen. Die Entscheidungsunterstützung insbesondere für präventive Zwecke ist zwar auch außerhalb von Pandemien ein Ziel der epidemiologischen Forschung, allerdings selten in einer solch dynamischen Situation. Zudem ist gegenwärtig die gesamte Bevölkerung direkt oder indirekt betroffen und variierende Maßnahmen zur Kontaktbeschränkung ändern die Expositionslage laufend. Entsprechend kommt auch der Kommunikation von epidemiologischen Forschungsmethoden und -ergebnissen eine besondere Bedeutung zu.

Dieser Artikel gibt einen Überblick über epidemiologische Ansätze in der Forschung zu COVID-19. Der Fokus liegt auf dabei auf wissenschaftlichen Fragen, zu deren Beantwortung Primärdaten aus Beobachtungsstudien oder Sekundärdaten mit Bezug zu COVID-19 genutzt werden können. Fragen der klinisch-epidemiologischen Forschung, z. B. zu neuen Therapien von COVID-19, werden nicht adressiert. In der Pandemie häufig verwendete epidemiologische Maßzahlen werden an anderer Stelle diskutiert (z. B. [1, 2]; siehe auch Priesemann et al. in diesem Heft) und daher in diesem Beitrag nicht behandelt.

Wir strukturieren den Beitrag mittels bevölkerungsbezogener Fragestellungen, die die verschiedenen Phasen des Verlaufs einer SARS-CoV-2-Infektion und COVID-19-Erkrankung auf Bevölkerungsebene betreffen. In der ersten Phase vor der Infektion beeinflussen individuelle und strukturelle Risikofaktoren das Infektionsrisiko und primärpräventive Maßnahmen können ihre Wirkung entfalten. Auf die Infektionsphase folgen die Symptomphase und anschließend – in einigen Fällen – die Hospitalisierungsphase, ggf. mit Intensivpflicht. Aus allen Phasen nach der Infektion ist jeweils Genesung oder auch Tod als Phasenaustritt denkbar. Nach der Akuterkrankung mit oder ohne Hospitalisierung schließt sich die Rehabilitationsphase an, in der Spätfolgen auftreten können – ggf. sogar noch weit nach dieser Phase. Die Frage nach möglichen Spätfolgen stellt sich nicht nur bei der Erkrankung, sondern auch im Kontext der Impfung gegen COVID-19. Wir gehen in diesem Beitrag jedoch nur kurz auf Forschungen zu Impffolgen ein.

In jeder dieser Phasen und den Übergängen zwischen ihnen gibt es eine Vielzahl von wissenschaftlichen Problemen, zu deren Lösungen die Epidemiologie beitragen kann. Dieser Beitrag beschreibt vorrangig die Situation in Deutschland und adressiert ausgewählte Fragestellungen, für die bereits epidemiologische Forschungsansätze und Datenquellen existieren oder erarbeitet werden. Es folgt ein Fazit, das Rahmenbedingungen der epidemiologischen Forschung in der COVID-19-Pandemie diskutiert und auf neue Entwicklungen hinweist.

## Methoden

Ausgangspunkt des methodischen Vorgehens war eine Diskussion der Autor:innen über Entwicklungsstand und Lücken der epidemiologischen Forschung in Deutschland. Wir führten anschließend in der Literaturdatenbank PubMed Recherchen mit Schlüsselwörtern zu epidemiologischem Forschungsdesign, verbunden mit Schlüsselwörtern zu COVID-19 und SARS-CoV‑2 und dem lokalisierenden Schlüsselwort „Germany“ durch. Rechercheergebnisse wurden nach Eignung für die vorab festgelegte inhaltliche Struktur durchgeschaut und in den passenden Textabschnitten zusammenfassend berichtet.

## Epidemiologische Ansätze zur Prävention durch Identifizierung von Risikogruppen

Zahlreiche Studien haben sich mit der Frage beschäftigt, welche Bevölkerungsgruppen ein besonders hohes Risiko für schwere Verläufe von COVID-19 oder sogar Tod haben. Schwere Krankheitsverläufe treten gehäuft bei älteren oder multimorbiden Patient:innen auf, insbesondere wenn chronisch obstruktive Lungenerkrankungen, Asthma, Nierenerkrankungen, Bluthochdruck, Diabetes mellitus oder Adipositas vorliegen. Diese Ergebnisse unterstützten Entscheidungen für den Schutz dieser Gruppen und für die Impfreihenfolge [3–5]. Für die Analyse entsprechender Kohorten konnten schon frühzeitig klinische Routinedaten genutzt werden. Am Beispiel der Analyse von klinischen Daten des National Health Service (NHS) zeigen sich jedoch auch Fallstricke bei der Nutzung dieser Daten, etwa wenn Komorbiditäten nicht angemessen berücksichtigt werden und so z. B. Rauchen fälschlicherweise als schützend für einen COVID-19-assoziierten Todesfall interpretiert wird [4].

Um aber die Frage zu beantworten, welche Bevölkerungsgruppen überhaupt ein erhöhtes SARS-CoV-2-Infektionsrisiko haben, werden bevölkerungsbasierte Studien benötigt. Hier geben Querschnittsdaten der positiv auf SARS-CoV‑2 getesteten Personen, wie sie täglich vom Robert Koch-Institut (RKI) veröffentlicht werden, erste wichtige Hinweise z. B. zur Alters- und Geschlechtsverteilung sowie zum zeitlichen und regionalen Verlauf und der Virusausbreitung [6]. Dies genügt aber nicht, um gezielte Schutzmaßnahmen zur Verringerung der Übertragungsraten einleiten zu können; denn dazu muss man u. a. die Orte und Personengruppen identifizieren, an bzw. bei denen besonders häufig Ansteckungen erfolgen. Neben der Analyse von Selbstangaben im Rahmen der Testung ist die Auswertung von Bewegungsdaten aus Mobiltelefonen ein eher neuer Ansatz im Kontext der epidemiologischen Forschung. Sie kann erste wichtige Hinweise auf typische Begegnungsorte liefern, aus denen dann Übertragungsorte abgeleitet werden können. Die Modellierung solcher Bewegungsdaten deutet z. B. darauf hin, dass der überwiegende Anteil an Ansteckungen vermutlich in Restaurants, Hotels, Fitnessstudios und Lebensmittelgeschäften erfolgt. Zudem zeigt sich, dass sich sozial benachteiligte Gruppen, die ihre Mobilität während der Pandemie weniger einschränken konnten, häufiger anstecken [7]. Für die Identifizierung tatsächlicher Übertragungssettings wären jedoch erweiterte digitale Contact-Tracing-Apps oder alternativ eine höchst detaillierte „manuelle“ Kontaktnachverfolgung notwendig – die Letztere erscheint jedoch bei hohen Infektionszahlen nicht praktikabel.

## Epidemiologische Feldstudien in der frühen Pandemiephase

Allerdings bedürfen die Schlussfolgerungen aus diesen Modellen einer Validierung durch reale Daten, wie sie nur epidemiologische Feldstudien generieren können. Dabei ist darauf zu achten, dass die benötigten Studien nicht nur symptomatische oder hospitalisierte Erkrankte einbeziehen. Stattdessen sollte innerhalb der Studienpopulation eine repräsentative Stichprobe aller positiv auf SARS-CoV‑2 Getesteten bezüglich soziodemografischer Charakteristika, Wohnsituation, Lebensstil, Freizeit- und Reiseverhalten sowie ihres beruflichen oder schulischen Umfelds mit einer geeigneten Kontrollgruppe verglichen werden. Die hierfür oft geforderten längsschnittlichen Kohortendesigns sind allerdings weniger geeignet, solange die Inzidenz noch niedrig ist. Aber gerade in der Anfangsphase einer Pandemie, wenn ein Ausbruch noch durch gezielte Maßnahmen eindämmbar ist, werden belastbare Daten zu den Ansteckungswegen benötigt. In der frühen Phase einer Pandemie sind Fallkontrollstudien das am besten geeignete analytische Studiendesign. Sobald jedoch Maßnahmen zur Kontaktbeschränkung greifen, verlieren diese Studien an Aussagekraft bzw. Allgemeingültigkeit, da sich die Expositionslage wesentlich verändert. Es ist daher sehr erstaunlich, dass in der frühen Phase der COVID-19-Pandemie kaum Fallkontrollstudien durchgeführt bzw. publiziert wurden. Eine mexikanische Fallkontrollstudie berichtete u. a. über erhöhte Infektions- bzw. Hospitalisierungsrisiken bei Hypertoniker:innen, Diabetiker:innen, Adipösen, Indigenen sowie bei Männern und Älteren. Allerdings fehlten grundlegende Daten und eine Unterscheidung zwischen Infizierten mit und ohne Symptome, sodass diese Studie wenig aufschlussreich blieb [8]. Auch eine US-amerikanische Fallkontrollstudie hilft kaum weiter, weil sie nur hospitalisierte Fälle einschloss und nur auf Unterschiede zwischen ethnischen Gruppen abzielte, ohne genau Aufschluss über Ansteckungswege zu geben [9]. Eine kleine chinesische Fallkontrollstudie suchte gezielt nach Lebensstilfaktoren und beobachtete dabei ein erhöhtes COVID-19-Hospitalisierungsrisiko z. B. bei Personen mit Schlafmangel und mit hoher körperlicher Aktivität. Ein erniedrigtes Risiko hatten Personen mit guter Handhygiene, täglichem Obstverzehr und geringem Alkoholkonsum [10]. Auch diese Studie lässt sich jedoch nicht allgemein auf asymptomatische bzw. nichthospitalisierte Infizierte übertragen. Daher besteht weiterhin dringender Bedarf nach gut geplanten Studien, die potenzielle Risikofaktoren für eine Ansteckung erfassen, quantifizieren und in Relation zueinander setzen. Insofern bleibt zu hoffen, dass weitere Studien, wie z. B. die vom RKI seit Anfang 2021 durchgeführte Fallkontrollstudie CoViRiS (Corona-Virus Risiko- und Schutzfaktoren; [11]), zeitnah die benötigten Erkenntnisse hervorbringen.

## Infektionsphase: Wie lassen sich Erkenntnisse zu Häufigkeit und Verteilung der Infektion und COVID-19-Erkrankung gewinnen?

Besondere öffentliche Aufmerksamkeit in der Pandemie erfährt die deskriptive Epidemiologie der SARS-CoV-2-Infektion und der damit verbundenen (symptomatischen) COVID-19-Erkrankung. Dieses Kernthema wird auf der einen Seite von den täglichen Statistiken zu Neuinfektionen, Hospitalisierungen/Intensivbehandlungen und Sterbefällen bedient. Hier werden im Wesentlichen klassische Ansätze der Surveillance von Infektionskrankheiten auf Basis definierter und vorbestehender Datenflüsse genutzt. In Deutschland sind damit vornehmlich die Gesundheitsämter, zuständige Landesbehörden und das RKI als zentrale Akteure des Öffentlichen Gesundheitsdienstes (ÖGD) gesetzlich beauftragt. Auf der anderen Seite ist seit Beginn der Pandemie klar, dass angesichts der präsymptomatischen Phase und asymptomatischer Verläufe mit anlassbedingten Tests nur ein Teil des Infektionsgeschehens abgebildet werden kann. Hier sind bevölkerungsbezogene Stichprobentestungen zur Erfassung der gesamten Infektionshäufigkeit zu einem Stichtag bzw. wiederholte Testungen in einer definierten Kohorte ein wichtiger Ansatz, der in Deutschland verschiedentlich umgesetzt wurde. Dabei können sowohl akute Infektionen mittels Antigen- und Nukleinsäurenachweis (Reverse-Transkriptase-Polymerase-Kettenreaktion (RT-PCR), Vollgenomsequenzierung zur Differenzierung) als auch ein zurückliegender Infektionsbeginn durch Antikörpernachweis zum Einsatz kommen. Eine gemeinsam von RKI und dem Deutschen Institut für Wirtschaftsforschung (DIW) im Oktober 2020 gestartete Studie nutzt bestehende Studienstrukturen des Sozio-oekonomischen Panels (SOEP) zur Erfassung von serologischen und infektiologischen Parametern mittels Selbsttestung, gepaart mit Fragebogendaten u. a. zu medizinischen und sozialen Aspekten. Teilnehmende Personen werden auch in zukünftige Erhebungen eingeschlossen, sodass hier grundsätzlich eine Langzeitverlaufsbeobachtung möglich ist. Lokale Studien wurden an besonders betroffenen Orten durchgeführt, u. a. im ersten großen Cluster in Heinsberg [12]. Von großem Interesse ist die Altersgruppe der Kinder und Jugendlichen, insbesondere aufgrund der sensiblen Thematik der Schulschließungen bzw. Aufrechterhaltung des Unterrichts. Bei der prospektiven zweiarmigen Studie COVID-19 Surveillance in School KIDs (sKIDs) wurden in England im Sommer 2020 wöchentlich Nasenabstriche bei Schüler:innen und Beschäftigten in 131 Schulen durchgeführt, in einem Teil der Schulen wurden zudem zu 3 Zeitpunkten bis zum November 2020 Blutproben zur Testung auf Antikörper gegen SARS-CoV‑2 entnommen. In beiden Armen erfolgte zusätzlich eine Fragebogenerhebung [13]. Eine diesem Ansatz entsprechende bundesweite Studie gab es in Deutschland nicht, dagegen einige regionale Untersuchungen in München [14] sowie in anderen großen Städten (u. a. Hamburg, Berlin). Aus dem Vergleich der Daten von anlasslosen und anlassbedingten Testungen lässt sich auf die Dunkelziffer schließen. In einer bayerischen Studie wurde aus dem Vergleich der Antikörperseroprävalenz mit positiven SARS-CoV-2-Testergebnissen auf der Basis von Abstrichen ein Faktor 6 als Dunkelziffer bei Kindern und Jugendlichen berechnet [15]; dies entspricht etwa der Dunkelziffer, die in der Ausbruchsuntersuchung in Heinsberg [12] ermittelt wurde. Aus methodischer Sicht handelt es sich bei den genannten Untersuchungsansätzen um typische epidemiologische Studiendesigns wie Querschnitts- oder Kohortenstudien. Neue SARS-CoV-2-Kohortenstudien wurden in Deutschland bis April 2021 nicht aufgesetzt. Bestehende Kohorten wie die NAKO-Gesundheitsstudie (ehemals Nationale Kohorte (NAKO)) wurden in der akuten Phase der Pandemie nicht umfänglich für die Evidenzgenerierung hinsichtlich der Infektionshäufigkeit und des Symptom- bzw. Erkrankungsverlaufs einbezogen. Über eine NAKO-Zusatzbefragung zur seelischen Gesundheit wird in diesem Heft berichtet [16].

Digitale epidemiologische Ansätze werden insbesondere zum Monitoring von Symptomen mittels Apps [17] und zur Untersuchung von Mobilitätsmustern im Zusammenhang mit Lockdownmaßnahmen [18] eingesetzt. Das Potenzial dieser Vorgehensweisen für die epidemiologische Forschung ist bisher nur in geringem Maße erschlossen – so könnten z. B. im Rahmen von Kohortenstudien Bewegungsdaten oder die vermehrt eingesetzten Selbsttests in Studienkontexten digital erfasst und damit neue Wege der Expositionsermittlung und -datenerfassung umgesetzt werden.

## Symptom- und Hospitalisierungsphase: Welche Forschungsdaten stehen zur Verfügung?

Die Beschreibung schwerer COVID-19-Verlaufsformen ist insbesondere durch den Kontakt Erkrankter mit Einrichtungen der medizinischen Versorgung möglich. Für den Erstkontakt kann dabei auf Daten aus Notaufnahmen und Arztpraxen zurückgegriffen werden, wie es in Deutschland z. B. über das Notaufnahmeregister realisiert ist, das tagesaktuell versorgungsnahe Daten zusammenführt [19]. Hospitalisierung wird typischerweise ebenfalls abgebildet über Sekundärdaten der Krankenhäuser (z. B. ICD-10-code-basierte Surveillance schwerer akuter respiratorischer Infektionskrankheiten des RKI (ICOSARI)) oder aus dem nachgeschalteten Controlling‑, Qualitätssicherungs- oder Finanzierungsprozess (z. B. über Daten der Krankenkassen; [20]). Die frühe Einführung von neuen ICD-Codes für COVID-19 (z. B. U07.1!, U07.2!) begünstigt die Identifikation der betroffenen Versorgungsprozesse, allerdings gibt es noch einige Fragen, was die adäquate Verwendung dieses Codes im ambulanten Versorgungsbereich betrifft. Die Nutzung der Daten der gesetzlichen Krankenkassen für COVID-19-bezogene Fragestellungen beschränkt sich deshalb bisher im Wesentlichen auf hospitalisierte COVID-19-Fälle. So zeigte eine entsprechende Auswertung soziale Unterschiede in der Häufigkeit von COVID-19-bedingten Hospitalisierungen [21]. Bereits zu Beginn der Pandemie wurden regionale Engpässe im Bereich der intensivmedizinischen Versorgung mit dem Schwerpunkt der Beatmungsmedizin vorhergesehen. Die Fachgesellschaft der Intensivmedizin und das RKI haben frühzeitig das Intensivregister der Deutschen Interdisziplinären Vereinigung für Intensiv- und Notfallmedizin (DIVI) aufgebaut, ein Beispiel für die in der Pandemie erstmalig erprobte bundesweite Echtzeitversorgungsforschung. Als schwierig erwies sich der Aufbau von Kohorten aus Personen, die positiv auf SARS-CoV‑2 getestet wurden bzw. an COVID-19 erkrankt waren. Als eine Initiative des Netzwerks Universitätsmedizin (NUM) rekrutiert das Nationale Pandemie Kohorten Netz (NAPKON) Patient:innen in gemeinsame Studien. Die flächendeckende Rekrutierung entsprechender Personen außerhalb von Kliniken erfordert die Zusammenarbeit mit Gesundheitsämtern, was jedoch aufgrund der Arbeitsbelastung der Gesundheitsämter sowie rechtlicher Einschränkungen nur sehr begrenzt möglich war. Dementsprechend fehlt es an Daten zur Untersuchung der Häufigkeit und der Determinanten unterschiedlicher Krankheitsverläufe sowie zu Langzeitverläufen.

## Kontrolle der Infektions‑, Symptom- und Hospitalisierungsphase: Welche nichtpharmakologischen Maßnahmen sind wirksam?

Mit dem Fortschreiten der Infektionsausbreitung, aber auch dem zunehmenden Wissen über Erkrankungsverlauf und besonders betroffene Gruppen stellen sich sehr schnell Fragen nach Interventionsmaßnahmen und deren Wirksamkeit. Auch zu deren Beantwortung werden epidemiologische Methoden benötigt, sowohl bei pharmakologischen als auch bei nichtpharmakologischen Interventionen. Epidemiologisch begründete Erkenntnisse sind eine wichtige Grundlage evidenzbasierten Entscheidens für oder gegen bestimmte Maßnahmen. In der COVID-19-Pandemie geht es um individuelle Maßnahmen wie das Maskentragen ebenso wie um Kontaktbeschränkungen, Schließung öffentlicher Einrichtungen, Geschäfte usw. sowie das Pausieren von (Massen‑)Veranstaltungen. Zum Maskentragen als Beispiel für eine Intervention, deren Wirksamkeit von Interesse ist, wurden in dem systematischen Review von Chu et al. [22] zwar 172 Beobachtungsstudien identifiziert, darunter jedoch nicht eine einzige randomisierte klinische Studie; 44 Studien mit einem vergleichenden Ansatz konnten ausgewertet werden. Ende 2020 wurde eine dänische randomisierte kontrollierte Studie veröffentlicht, die in der Allgemeinbevölkerung das Tragen von chirurgischen Masken in Interventions- und Kontrollgruppe verglich [23]. Personen, die eine Empfehlung zum Maskentragen erhalten hatten, wiesen ein Odds Ratio von 0,82 (95 % Konfidenzintervall [KI] 0,54–1,23) bezüglich des Auftretens einer SARS-CoV-2-Infektion auf. In der Studie machten sich die Autor:innen eine besondere Situation in Dänemark zunutze, in der es zwar Empfehlungen zum Abstandhalten gab, das Maskentragen angesichts eines moderaten Infektionsgeschehens jedoch weder verbreitet noch empfohlen war. Anzumerken ist einschränkend, dass hier nur das individuelle Infektionsrisiko für die Maskentragenden selbst bewertet werden konnte. Dennoch ließ sich hier die real existierende Situation nutzen, um eine randomisierte Studie zu einer zusätzlichen Schutzmaßnahme – dem Maskentragen – durchzuführen. Für die Evaluation anderer Maßnahmen wie etwa Schulschließungen und Ausgangssperren boten sich dagegen bisher zumeist nur Ansätze an, die regionale Unterschiede oder unterschiedliche Zeitperioden der Maßnahmeneinführung für die Abschätzung der Wirksamkeit nutzten. Hierbei ergibt sich ein fließender Übergang zur Politikevaluation, die allerdings ebenfalls epidemiologisch untermauert ist. Eine Untersuchung von Mitze et al. [24] analysierte die Einführung der Maskenpflicht in 401 Kreisen in Deutschland, indem synthetische Kontrollen zu den „Interventionskreisen“ generiert wurden und das Auftreten von Infektionen über die Zeit verglichen wurde. Im Ergebnis zeigt die Studie eine Reduktion registrierter Infektionen um 15–75 % in den ersten 20 Tagen nach Einführung einer Maskenpflicht. Der ökologische Ansatz wird hier durch die Konstruktion der synthetischen, aus mehreren Vergleichskreisen berechneten Kontrollregionen verfeinert, aber die Ergebnisse sind aufgrund der grundsätzlichen methodischen Nachteile solcher Korrelationsstudien wenig belastbar. Weitere Ansätze der Interventionsforschung, etwa Zeitreihenanalysen oder länderübergreifende Modellierung von Infektionsdaten in Abhängigkeit vom zeitlichen Einsetzen bestimmter Maßnahmen, wurden ebenfalls eingesetzt, um die Wirkung von bevölkerungsbezogenen Interventionen zu untersuchen [25]. Hier zeigen sich vielfältige Überschneidungen epidemiologischer mit ökonometrischen, spezifischen statistischen und Künstliche-Intelligenz-Methoden, die auf ein interessantes Entwicklungsfeld für die epidemiologisch-interdisziplinäre Forschung verweisen.

## Kontrolle der Infektions‑, Symptom- und Hospitalisierungsphase: Welche Bedeutung haben indirekte Gesundheitsfolgen?

Neben den direkten gesundheitlichen Folgen einer SARS-CoV-2-Infektion sind mögliche indirekte Gesundheitsfolgen, die durch den Lockdown und die nachfolgenden Einschränkungen des Alltagslebens verursacht werden, von großer Relevanz für die Gesundheitsforschung. Dabei unterscheiden wir 2 Typen gesundheitlicher Begleitfolgen: mögliche psychosoziale Folgen aufgrund der Pandemie bzw. des Lockdowns und Folgen einer verspäteten oder nicht erfolgten Inanspruchnahme von medizinischen Versorgungsleistungen. In Bezug auf Auftreten, Diagnose und Behandlung anderer Erkrankungen als COVID-19 kann eine Analyse der Inanspruchnahme dieser Leistungen basierend auf Krankenkassenabrechnungsdaten sowie mittels Informationen aus Arztpraxen oder Krankenhäusern oder direkt anhand von Patientenbefragungen erfolgen. Bei Studien basierend auf Sekundärdaten ist allerdings Vorsicht geboten, da ggf. nicht unterschieden werden kann, ob bestimmte Leistungen nicht in Anspruch genommen wurden, weil den Versicherten diese Leistung z. B. aufgrund der pandemiebedingten Auslastung der Klinik nicht angeboten wurde oder weil sie diese Leistung aus Angst vor Ansteckung nicht in Anspruch genommen haben. Daher sollte eine solche Sekundärdatenanalyse durch eine Primärdatenerhebung in einer unselektierten Population ergänzt werden. Zu diesem Zweck bieten sich bereits etablierte Kohorten wie die NAKO-Gesundheitsstudie oder das SOEP an. Anhand geeigneter Befragungsinstrumente können so die möglichen gesundheitlichen Folgen von nicht oder verspätet wahrgenommenen ärztlichen und psychotherapeutischen Leistungen, aber auch von psychischen Belastungen der Bevölkerung [16] erfasst und bewertet werden.

Weltweit wurden zahlreiche Studien durchgeführt, um sowohl Informationen über Nichtinanspruchnahmen von Versorgungsleistungen und den daraus entstehenden gesundheitlichen Konsequenzen als auch zur psychischen Belastung verschiedener Bevölkerungsgruppen zusammenzutragen. Im Folgenden seien exemplarisch für verschiedene Studientypen 3 Studien aus Deutschland aufgeführt. Im Corona Snapshot Monitoring (COSMO; [26]) werden seit März 2020 Querschnittsstichproben von je ca. 1000 Personen im (2-)wöchigen Abstand u. a. zu ihrer Wahrnehmung der Pandemie und ihrer psychologischen Belastung online befragt. Am 9./10.02.2021 fand die 35. Befragung statt, bei der insgesamt 64 % (höchster Wert seit Beginn der Befragung) ihre persönliche Situation momentan als belastend angaben. Dieser Anteil war bei Jüngeren (< 30 Jahre) besonders ausgeprägt (68 %), allerdings wurde auch bei Älteren (> 65 Jahre) ein Anstieg beobachtet [27].

Vielfältige Veränderungen lassen sich in Auftreten und Versorgung akuter und chronischer Erkrankungen in der Pandemie erkennen: So zeigte eine Analyse von Abrechnungsdaten der Allgemeinen Ortskrankenkass(en) (AOK) eine reduzierte Anzahl von Appendektomien im Vergleich zu den gleichen Zeiträumen der beiden Vorjahre [28]. Dies betraf allerdings nur Fälle mit einfacher akuter oder nicht akuter Appendizitis, nicht jedoch Fälle mit komplizierter akuter Appendizitis. Die Autoren sahen in diesen Ergebnissen daher keinen Hinweis auf eine Verschlechterung der Behandlung von Appendizitiden.

Ein Behandlungsrückgang um 12,6 % im Vergleich zu den Märzmonaten der 3 Vorjahre zeigte sich bei 15.800 Patienten mit akutem Myokardinfarkt im März 2020, die an der Studie „Feedback Intervention and Treatment Times in ST-Elevation Myocardial Infarction“ (FITT-STEMI; [29]) teilnahmen. Klinisch wichtige Qualitätsindikatoren des STEMI-Managements, wie z. B. die Arzterstkontakt-Ballon-Zeit, wurden nicht beeinträchtigt. Die genannten Studien stehen exemplarisch für die Möglichkeiten, mit Routinedaten oder mit Daten aus laufenden Studien epidemiologisch bedeutsame Fragestellungen zu analysieren. So können auch Erkenntnisse zu einer möglichen Reduktion von Überbehandlungen ebenso wie zur Abnahme von unfallbedingten Traumata während der Pandemie gewonnen und für Public Health und die Weiterentwicklung der Versorgung genutzt werden.

## Impfung gegen COVID-19: Wie sind die Wirksamkeit und Sicherheit in Subgruppen?

Flächendeckende Impfungen gelten als das wichtigste Instrument, um Pandemien zu überwinden und schrittweise zu einem Normalzustand zurückzukehren. Mittlerweile wurden für mehrere COVID-19-Impfstoffe klinische Studien durchgeführt, die eine hohe Wirksamkeit und ein vertretbares Nebenwirkungsprofil aufzeigten [30, 31], sodass einige dieser Impfstoffe bereits eine Zulassung erhalten haben. Wie immer bei neuen Arzneimitteln sind jedoch zum Zeitpunkt der Zulassung noch nicht alle Fragen zur Wirksamkeit und Sicherheit beantwortet. Grundsätzlich ist deshalb der Aufbau von Datenbasen wichtig, die Beobachtungsstudien zeitnah nach der Zulassung ermöglichen. Bei den COVID-19-Impfungen kommt dem eine noch größere Bedeutung zu, da sie kurz nach der Zulassung großflächig bei Millionen von Menschen zur Anwendung kommen. Zu den offenen Fragen zählen die Langzeitsicherheit und -wirksamkeit der verschiedenen Impfstoffe in verschiedenen Subgruppen. In den Zulassungsstudien werden die Gruppen aus ethischen Gründen nach 6 Monaten aufgelöst, d. h., den Personen im Kontrollarm wird dann auch die Impfung angeboten. Die Fragen zur Langzeitsicherheit und -wirksamkeit werden somit nicht durch Studien mit randomisiert kontrolliertem Design adressiert und sind nur durch Beobachtungsstudien zu klären. Die Abrechnungsdaten gesetzlicher Krankenversicherungen stellen eine wertvolle Datengrundlage für Beobachtungsstudien nach der Zulassung von Arzneimitteln dar und könnten in der aktuellen Situation für die Untersuchung der COVID-19-Impfungen in idealer Weise genutzt werden. Eine zentrale Voraussetzung dafür wäre allerdings, dass der jeweils verabreichte Impfstoff, wie es bei verschreibungspflichtigen Medikamenten üblich ist, in den Kassendaten erfasst wird, sodass der Expositionsstatus und -zeitpunkt ermittelt und dann zu später auftretenden Erkrankungen in Beziehung gesetzt werden können. Dies ist für COVID-19-Impfungen bisher jedoch nicht der Fall, da sie nicht über die Kassen abgerechnet werden. In der „Nationalen Impfstrategie COVID-19“ ist zwar angemerkt, dass eine Auswertung von Leistungs- und Abrechnungsdaten zur Sicherheit von COVID-19-Impfstoffen durchgeführt werden soll [32], doch wurden in der Corona-Impfverordnung nicht die notwendigen Voraussetzungen geschaffen, um die Informationen zur Impfung zeitnah und mit hoher Trefferquote mit Kassendaten verknüpfen zu können. So ist rechtlich weder die Erfassung und Weiterleitung der Versichertennummer noch der Versichertenzugehörigkeit durch die Impfzentren vorgesehen. Seitens der epidemiologischen Fachgesellschaften wurde auf diese Defizite hingewiesen. Es wurde außerdem aufgezeigt, wie es gelingen kann, Geimpfte und Nichtgeimpfte hinsichtlich schwerwiegender Ereignisse basierend auf Versichertendaten zeitnah zu vergleichen, was aber keine Berücksichtigung fand. Somit ist davon auszugehen, dass in Deutschland in absehbarer Zeit keine brauchbare Datenbasis zur Verfügung stehen wird, um die o. g. Fragen adäquat zu adressieren bzw. Verdachtsfällen, die durch das Spontanmeldesystem generiert werden, mit einem geeigneten Studiendesign zeitnah nachzugehen.

## Schlussfolgerungen

Mit Beginn der COVID-19-Pandemie Anfang 2020 wurden besondere Anforderungen und Erwartungen an die Epidemiologie gestellt. Es mussten unter hohem Zeitdruck umfassende und differenzierte deskriptive Daten ebenso wie genaue analytische Erkenntnisse erarbeitet werden. Eine derartige Situation birgt Risiken und Chancen. Vorhandene Infrastrukturen der Gesundheitsberichterstattung und des Datenaustausches zwischen Gesundheitsämtern sowie Universitäten und Forschungseinrichtungen mussten sich sehr schnell auf die Bearbeitung epidemiologischer Fragestellungen mit Bedeutung für das Management und Verständnis der Pandemie einstellen. Hierfür stand das gesamte Instrumentarium der deskriptiven und analytischen Epidemiologie zur Verfügung, ergänzt durch Ansätze der digitalen Epidemiologie, die sowohl in der zeitnahen Beschreibung der regionalen und internationalen Infektionsausbreitung als auch z. B. bei der Analyse von Mobilitätsmustern genutzt werden konnten. Als Datenquellen für die Analyse der klinischen Phasen der COVID-19-Erkrankungen kommen zudem Sekundärdaten der Krankenkassen zum Einsatz, wobei die Nutzbarkeit der Daten aus den ambulanten Kassendaten zu COVID-19 noch fraglich ist; das Potenzial von Krankenkassendaten in Bezug auf Fragestellungen zur Impfung kann voraussichtlich kaum genutzt werden.

Die Vielzahl der Fragestellungen, von denen wir – orientiert an den Phasen des Infektions- und Erkrankungsverlaufs – einige exemplarisch in diesem Beitrag ansprechen, bedingt eine ebenso große Spannbreite bei den Forschungsdesigns und Datenquellen. Grundsätzlich ergeben sich hier Fragen nach der Koordination von Forschung. Anstrengungen etwa seitens des RKI oder im Rahmen der Nationalen Forschungsdateninfrastruktur für personenbezogene Gesundheitsdaten (NFDI4Health) setzen genau hier an. Dennoch wird sich im Nachgang der Pandemie eine kritische Analyse der epidemiologischen Forschung mit der effektiven Vermeidung von Research Waste, also „Forschungsmüll“ in einer solch dynamischen Situation beschäftigen müssen. Zu diskutieren ist, an welchen Stellen eine effektivere Nutzung bereits vorhandener Gesundheitsdaten, z. B. durch Einbeziehung von Gesundheitsämtern und die Verknüpfung verschiedener Datenquellen wie Impfdaten und Krankenkassendaten, gefordert werden muss.

## Fazit

Um den Beitrag der Epidemiologie zur Pandemiebewältigung zu stärken, müssen übergreifende Forschungsstrukturen verbessert und eine frühzeitige Abstimmung für die Nutzung vorhandener gesundheitsbezogener Daten erfolgen. Ein Ad-hoc-Aufbau solcher Strukturen ist allerdings kaum zu leisten, daher musste in der aktuellen Situation kreativ mit vorhandenen Forschungs- und Datenstrukturen gearbeitet werden. Zur Beantwortung vieler Forschungsfragen, etwa zur Wirksamkeit von nichtpharmakologischen Interventionen, konnten epidemiologisch Forschende aus Deutschland daher nur wenig beitragen – zumindest keine Primärforschung. Das heißt, im Fall zukünftiger Pandemien müssen bereits Konzepte und Strukturen etabliert sein, die die disziplinübergreifende Nutzung von vorhandenen Datenquellen, wie etwa Abrechnungsdaten der Krankenkassen und von existierenden Kohorten, wie etwa die NAKO-Gesundheitsstudie, ermöglichen.

Ein weiterer Erkenntnisgewinn ist, dass Forschungsdaten zukünftig frühzeitig und standardisiert im Sinne von Open Data auf gut zugänglichen Plattformen zur Verfügung gestellt werden müssen. Dies wird eine erhebliche Hilfestellung bei der Bewältigung vergleichbarer Herausforderungen sein. Hier geht es um Data Preparedness, also eine gute Vorbereitung von Forschungsdaten und -infrastrukturen auf Krisensituationen. Die hierfür notwendige Zusammenarbeit der epidemiologisch Forschenden wird sich ohne wesentliche Hindernisse organisieren lassen, denn schon in der aktuellen Pandemie gibt es viel kollegiale Abstimmung, etwa im Rahmen des Kompetenznetzes Public Health zu COVID-19, im Aufbau von Forschungsdateninfrastrukturen wie der NFDI4Health oder dem NUM – auch dies eine positive Erkenntnis im Rahmen der Pandemie.
